# CD18 and CD36 expression in neutrophils from tumors and tumor-draining lymph nodes: implications for metastasis in oral squamous cell carcinoma

**DOI:** 10.1007/s10585-025-10356-z

**Published:** 2025-06-23

**Authors:** Sandra Ekstedt, Eduardo I. Cardenas, Krzysztof Piersiala, Vilma Liljeström, Marianne Petro, Monika Ezerskyte, Pedro Farrajota Neves da Silva, Susanna Kumlien Georén, Lars-Olaf Cardell

**Affiliations:** 1 Division of ENT Diseases, Department of Clinical Sciences, Intervention and Technology, Karolinska Institutet, Stockholm, Sweden; 2 Department of Otorhinolaryngology, Karolinska University Hospital, Stockholm, Sweden; 3 Department of Pathology and Cytology, Karolinska University Hospital, Stockholm, Sweden

**Keywords:** Neutrophils, Cancer, Metastasis, Lymph nodes, CD18, CD36, OSCC

## Abstract

**Background:**

Neutrophil infiltration in tumors and tumor-draining lymph nodes (TDLNs) influences oral squamous cell carcinoma (OSCC) progression and metastasis. Neutrophils can exhibit an immunosuppressive phenotype, with CD18 and CD36 potentially linked to this. This study characterizes CD18/CD36 expression on neutrophils from different OSCC microenvironments and their association with metastasis.

**Methods:**

We assessed CD18 and CD36 expression on neutrophils from OSCC tumors, TDLNs, and healthy lymph nodes using flow cytometry. We also examined whether co-culture with the CAL27 oral cancer cell line influenced CD18/CD36 expression in blood neutrophils from healthy donors.

**Results:**

Neutrophils from OSCC tumors and TDLNs exhibited higher CD18 expression than those from healthy lymph nodes, while CD36 was increased only in OSCC tumors. The highest CD18/CD36 expression was observed in metastasis. In vitro co-culture with CAL27 cells prolonged neutrophil survival and enhanced CD18 expression but had no impact on CD36 levels.

**Conclusion:**

Increased CD18/CD36 expression in OSCC neutrophils, particularly in metastasis, suggests their role in tumorigenesis. The elevated CD18 expression in TDLNs highlights enhanced neutrophil-lymphocyte interactions during cancer progression. Our in vitro findings underscore the ability of cancer cells to modulate neutrophil lifespan and phenotype, though this may not fully replicate the tumor microenvironment. This study provides insight into neutrophil contributions to OSCC progression and supports their potential as therapeutic targets.

**Supplementary Information:**

The online version contains supplementary material available at 10.1007/s10585-025-10356-z.

## Introduction

Oral squamous cell carcinoma (OSCC) is the most prevalent form of head and neck SCC (HNSCC), with its incidence seemingly on the rise, particularly among younger individuals [1]. Current standard of care (SOC) treatment for these patients involves invasive surgical resection followed by adjuvant radiation and/or chemoradiation that can only offer a five-year overall survival rate of approximately 50% [2]. Furthermore, this type of treatment often results in severe and permanent side effects such as impaired speech and swallowing difficulties. For these reasons, cancer immunotherapy has emerged as a promising alternative to traditional approaches in the management of OSCC. Indeed, blockade of the immune checkpoint molecule PD-1 has been used to prolong the survival of patients with advanced stages of HNSCC [3]. Nevertheless, challenges persist, with many patients failing to respond adequately or experiencing only marginal improvements.

A significant hurdle in advancing cancer immunotherapy lies in the intricate immunosuppressive milieu that surrounds tumors, which has prompted a predominant focus on the tumor microenvironment. However, disruptions in tumor-draining lymph nodes (TDLNs also called sentinel nodes) can also compromise anti-tumor immunity and thus limit the response to cancer immunotherapy. In agreement with this, lymph node metastasis serves as a robust prognostic indicator in head and neck cancer, with metastasis in a single lymph node reducing 3-year overall survival by 30% [4, 5]. Interestingly, accumulation of neutrophils in tumors and TDLNs has emerged as a marker of poor prognosis across different cancer types, including head and neck cancers [6–9].

Traditionally viewed as simple, homogenous, and short-lived, neutrophils are now known to display various phenotypes and functions in cancer. For instance, a recent study indicates that a subset of neutrophils displaying antigen-presenting markers such as HLA-DR, CD80, and CD86 can transport tumor antigens into TDLNs during early stages of head and neck cancer, thereby initiating a tumor-specific immune response [9]. On the other hand, a different subset of neutrophils known as polymorphonuclear myeloid-derived suppressor cells (PMN-MDSCs) displays potent immunosuppressive properties in advanced stages of cancer [9].

PMN-MDSCs hinder anti-cancer T cell responses through mechanisms, such as immune checkpoint ligand expression (e.g., PD-L1), amino acid depletion, and production of reactive oxygen species (ROS) [10]. Notably, PMN-MDSCs appear to exert at least some of these immunosuppressive functions via CD18-dependent contact with lymphocytes [11]. Nevertheless, a lack of established surface markers for PMN-MDSCs makes their identification difficult. To date, PMN-MDSCs are typically identified via gradient centrifugation by their characteristic low density or via functional tests to prove their immunosuppressive potential. Although recent studies suggest that PMN-MDSCs have a higher expression of lipid transport proteins such as CD36 [12, 13], little is known regarding the expression of these and other neutrophil markers at different anatomical sites relevant to cancer (e.g., tumor, TDLN, etc.), as well as their association with metastasis.

In this study, we characterized the expression of CD18 and CD36 in neutrophils across different compartments: tumors and TDLNs from patients with metastatic and non-metastatic OSCC, as well as lymph nodes from healthy individuals. Additionally, we investigated the impact of in vitro culture with CAL27 cells—an OSCC cell line—on these neutrophil markers. Understanding how these markers are influenced by different microenvironments and direct interaction with cancer cells offers critical insights into their potential roles in OSCC progression.

## Materials and methods

### Ethical compliance

This study was conducted in adherence to ethical principles outlined by the 1964 Helsinki Declaration and its subsequent amendments or equivalent ethical standards. Prior to participation, informed consent was secured from every individual included in the study. The research received approval from the Local Ethics Committee (Approval No.: 2019–03518).

### Patient characteristics

This study included 39 patients (Table 1) with OSCC, with a mean age of 67.8 years (± 10.8) and a slight male predominance (53.8%). Smoking history was almost evenly distributed between never smokers (46.2%) and current/previous smokers (53.8%). Most tumors were early-stage (pT1-2, 61.5%) and node-negative (pN0, 71.8%). The mobile tongue was the most common tumor site (61.5%), followed by the gingiva (23.1%). Recurrence occurred in 30.8% of patients. The study cohort included patients with OSCC located in various oral cavity subsites, including the mobile tongue (*n* = 24), gingiva (*n* = 9), floor of the mouth (*n* = 3), and buccal mucosa (*n* = 3). Stratification based on tumor location was not performed, as the primary objective was to characterize neutrophil phenotypes within the broader category of HPV-negative head and neck squamous cell carcinoma (HNSCC), rather than to assess site-specific immunological differences between specific anatomical sites.

**Table 1:** Demographic characteristics and clinicopathological data of enrolled patients

Variable	*N* (%)
**Age (mean ± SD)**	67.8 ± 10.8
**Sex**	
Female	18 (46.2)
Male	21 (53.8)
**Smoking history**	
Never smoker	18 (46.2)
Previous/current smoker	21 (53.8)
**pT status**	
pT1	13 (33.3)
pT2	11 (28.2)
pT3	8 (20.5)
pT4	7 (18.0)
**pN status**	
N0	28 (71.8)
N+	11 (28.2)
**Tumour site**	
Mobile tongue	24 (61.5)
Gingiva	9 (23.1)
Floor of the mouth	3 (7.7)
Buccal mucosa	3 (7.7)
**Recurrence status**	
Recurrence-free	27 (69.2)
Recurrence	12 (30.8)

Eligibility for the study was restricted to individuals diagnosed with primary or recurrent OSCC, who were scheduled for tumor with selective neck dissection or recurrence excision. In this procedure, TDLNs were identified using SPECT-CT and confirmed intraoperatively with a gamma probe at Karolinska University Hospital, Stockholm, Sweden. The pathologys digonsed the TDLN as metastatic or non-metastatic For a comprehensive explanation of the TDLN identification procedure utilized at Karolinska University Hospital, see Kågedal et al. [14].

Exclusion criteria included any systemic autoimmune disease, secondary malignancies, secondary malignancies or a history of hemo-lymphopoietic cancers, on-going infection and any acute or chronic conditions that could potentially alter the immunological landscape within the lymph nodes. Furthermore, we collected neck lymph nodes from individuals undergoing submandibular gland excision due to sialolithiasis and designated them as “healthy lymph nodes” (hLN), ensuring these subjects did not fulfill any exclusion criteria.

### Aim, design, and setting of the study

The aim of this study was to investigate the expression levels of CD18 and CD36 on neutrophils in various compartments associated with OSCC and explore their potential link to metastatic processes. This research was designed as an observational study, analyzing tissue samples and neutrophils from OSCC patients and healthy donors. It was conducted at Karolinska University Hospital, Stockholm, Sweden, where tumor and lymph node samples were collected during tumor resection with selective neck dissection for OSCC patients or during planned surgery of patients without cancer. Neutrophil co-culture experiments were also carried out in vitro using CAL27 to further investigate their interactions. CAL27 (RRID: CVCL_1107) cells have been authenticated using SNP profiling within the last three years and all experiments were performed with mycoplasma-free cells.

### Tissue preparation for neutrophil analyses

After surgery, the unfixed tissues were immediately transported to the Pathology Department, where pathologists processed the samples to provide a portion of the tumor and lymph nodes for analysis. The received tissue samples were stored in pre-chilled MACS Tissue Storage Solution (Miltenyi, Bergisch Gladbach, Germany). The samples were then passed through a 100 μm cell strainer (BD Biosciences #352360) and rinsed with RPMI-1640 (Invivogen, San Diego, CA, USA) to generate single-cell suspensions.

### Neutrophil isolation and culture

Whole blood from five healthy donors was obtained at the Blood Donor Center (Karolinska University Hospital, Solna) and neutrophils were isolated using the MACSxpress Whole Blood Neutrophil isolation kit and MACSxpress Erythrocyte Depletion kit (both from Miltenyi, Bergisch Gladbach, Germany). Isolated neutrophils were cultured (37 °C, 5% CO_2_) alone or together with CAL27 cells for 3, 24, 48, or 72 h, or 6 days in DMEM culture media containing 1% penicillin-streptomycin and 10% fetal bovine serum (all from Gibco). All CAL27 cells used in the study were between passage 6 and 10.

### Flow cytometry

Single-cell suspensions derived from lymph node tissues were Fc-blocked, stained with CD15, CD16, CD18, CD36 and CD45 (all from BD Biosciences), and fixed (1% paraformaldehyde in PBS) prior to analysis. Cultured neutrophils were washed in PBS, Fc-blocked, and stained with antibodies against CD15, CD18, and CD36 (all from BD Biosciences, Supplementary Table 1) prior to analysis. Of note, viability of cultured neutrophils was determined using a commercially available apoptosis detection kit (eBioscience, Carlsbad, CA, USA), which uses propidium iodide (PI) and annexin V to identify dead and apoptotic cells, respectively, and precludes the use of fixative agents. Flow cytometry was performed with BD LSR FORTESSA x20 (RRID: SCR_025285) and the resulting data was analyzed using FlowJo version 10.7.1 (RRID: SCR_008520) (both from BD Biosciences). The gating strategy for neutrophil identification is summarized in Supplementary material Figs. 1 and 2.

### Statistical analysis

Statistical analyses were performed using GraphPad Prism software (RRID: SCR_002798)version 10.0, GraphPad Software, La Jolla, CA). All data are presented as mean ± SEM. A p-value of < 0.05 was considered statistically significant (*P < 0.05, **P < 0.01, *P < 0.001). A D’Agostino & Pearson omnibus normality test was used to assess the normality of the data distribution. For comparisons between more than two groups, a one-way ANOVA with Bonferroni’s post-hoc test was performed. For comparisons involving two independent variables, a two-way ANOVA followed by Bonferroni’s post-hoc test was used. For comparisons between two groups, a paired Student’s t-test was performed.

## Results

### Elevated CD18 and CD36 levels in neutrophils from OSCC-associated tissues

Neutrophils infiltrating tumor and TDLNs exhibit distinct phenotypes that may significantly influence cancer progression. To elucidate the role of neutrophils in metastasis, the surface expression of CD18 and CD36 was quantified on neutrophils isolated from tumors and TDLNs of patients with OSCC and lymph nodes from healthy donors.

**Fig. 1:**
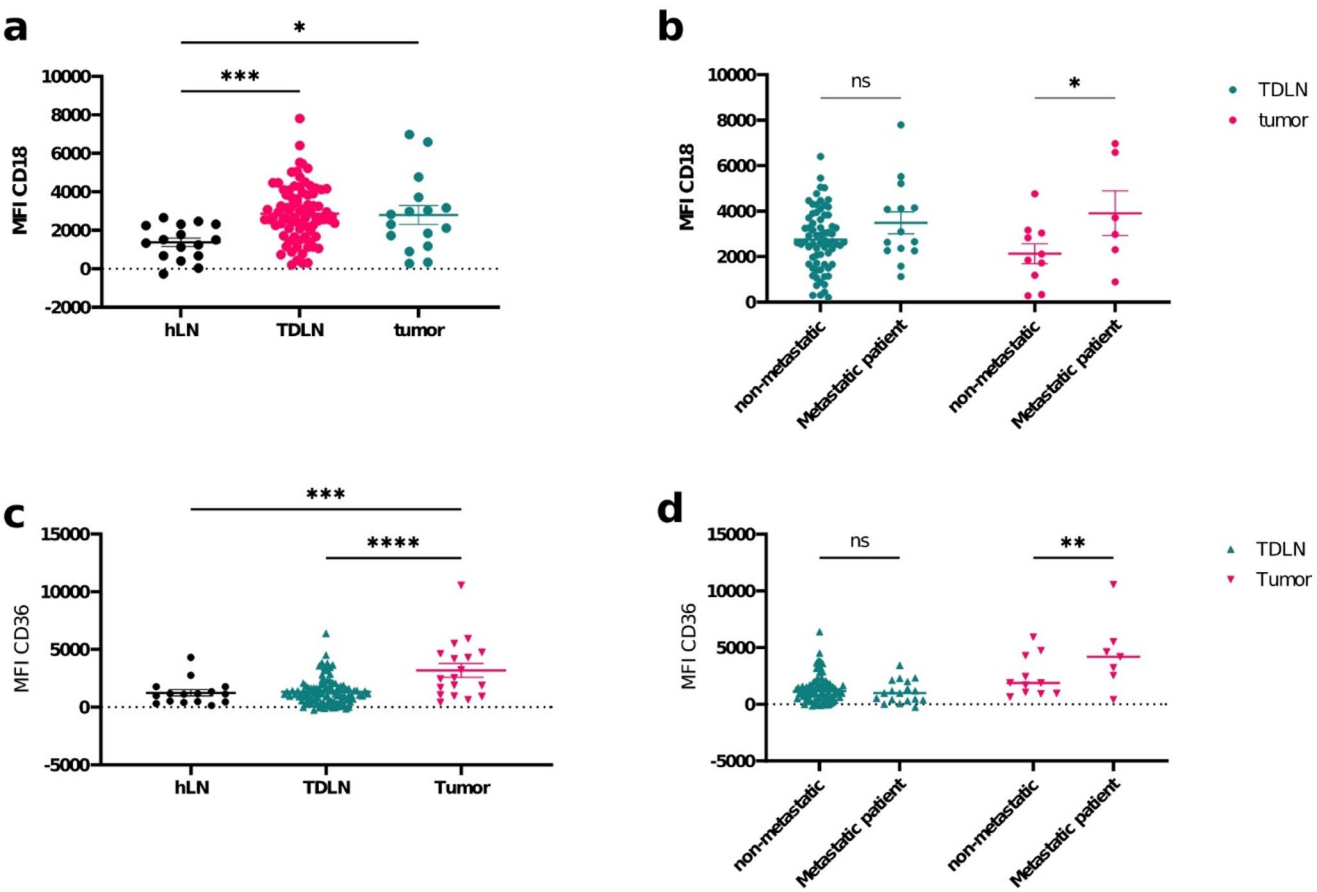
Differential Expression of CD18 and CD36 Across Tumor and Lymph Node Microenvironments. **(a**,** c)** Comparison of **(a)** CD18 and **(C)** CD36 expression in healthy lymph nodes (hLN, *n* = 16), tumor-draining lymph nodes (TDLNs, n_CD18_ = 84 and n_CD36_ = 105), and tumor tissues (n_CD18_ = 16 and n_CD36_ = 18) performed by one-way ANOVA followed by Bonferroni’s post-hoc test. **(b**,** d)** Comparison of the expression of **(b)** CD18 and **(d)** CD36 in neutrophils from TDLNs and tumor tissues from patients with and without metastasis performed by unpaired two-way ANOVA followed by Bonferroni’s post-hoc test. Data across all panels are presented as mean ± SEM, with statistical significance indicated by *p < 0.05, **p < 0.01, ***p < 0.001, ****p < 0.0001

Neutrophils from OSCC tumors and TDLNs showed significantly elevated levels of CD18 compared to neutrophils from hLNs (Fig. 1a; *p* = 0.0216 and *p* = 0.0009, respectively)suggesting their potential role in facilitating immune cell interactions during metastasis. This was further supported by the finding that CD18 expression was elevated in neutrophils from metastatic OSCC tumors than in neutrophils from non-metastatic tumors, indicating a possible involvement of CD18 in the metastatic processes (Fig. 1b; *p* = 0.0450). In addition, neutrophils from pathologically confirmed metastatic TDLNs showed the same indication, though unsignificant (Fig. 1b, *p* = 0,1821).

Interestingly, neutrophils from OSCC tumors but not from TDLNs exhibited significantly higher levels of CD36 compared to those from hLNs (Fig. 1c; *p* = 0.0001 and *p* = 0.0002 respectively). Levels of CD36 in neutrophils from TDLNs were similar to hLN and significantly lower than in tumor. This elevated expression of CD36 in tumor-associated neutrophils may reflect an adaptive mechanism linked to tumor-cell interactions, such as lipid metabolism or immune suppression within the tumor microenvironment. Moreover, CD36 levels were notably higher in neutrophils from metastatic OSCC tumors compared to non-metastatic tumors (Fig. 1d; *p* = 0.0049), suggesting that CD36 might contribute to the unique metabolic demands or immune evasion strategies of metastatic tumor sites.

### Impact of CAL27 co-culture on neutrophil lifespan and CD18 expression

**Fig. 2:**
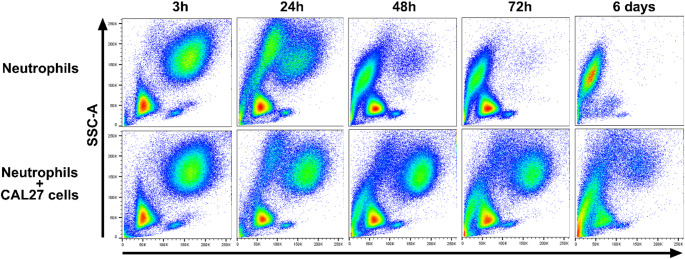
Representative flow cytometry plots of cultured neutrophils. Blood neutrophils from 5 healthy donors were cultured in vitro alone or together with CAL27 cells for 3, 24, 48, and 72 h (h), as well as 6 days. cultured in vitro alone or together with CAL27 cells for 3, 24, 48, and 72 h (h), as well as 6 days

**Fig. 3:**
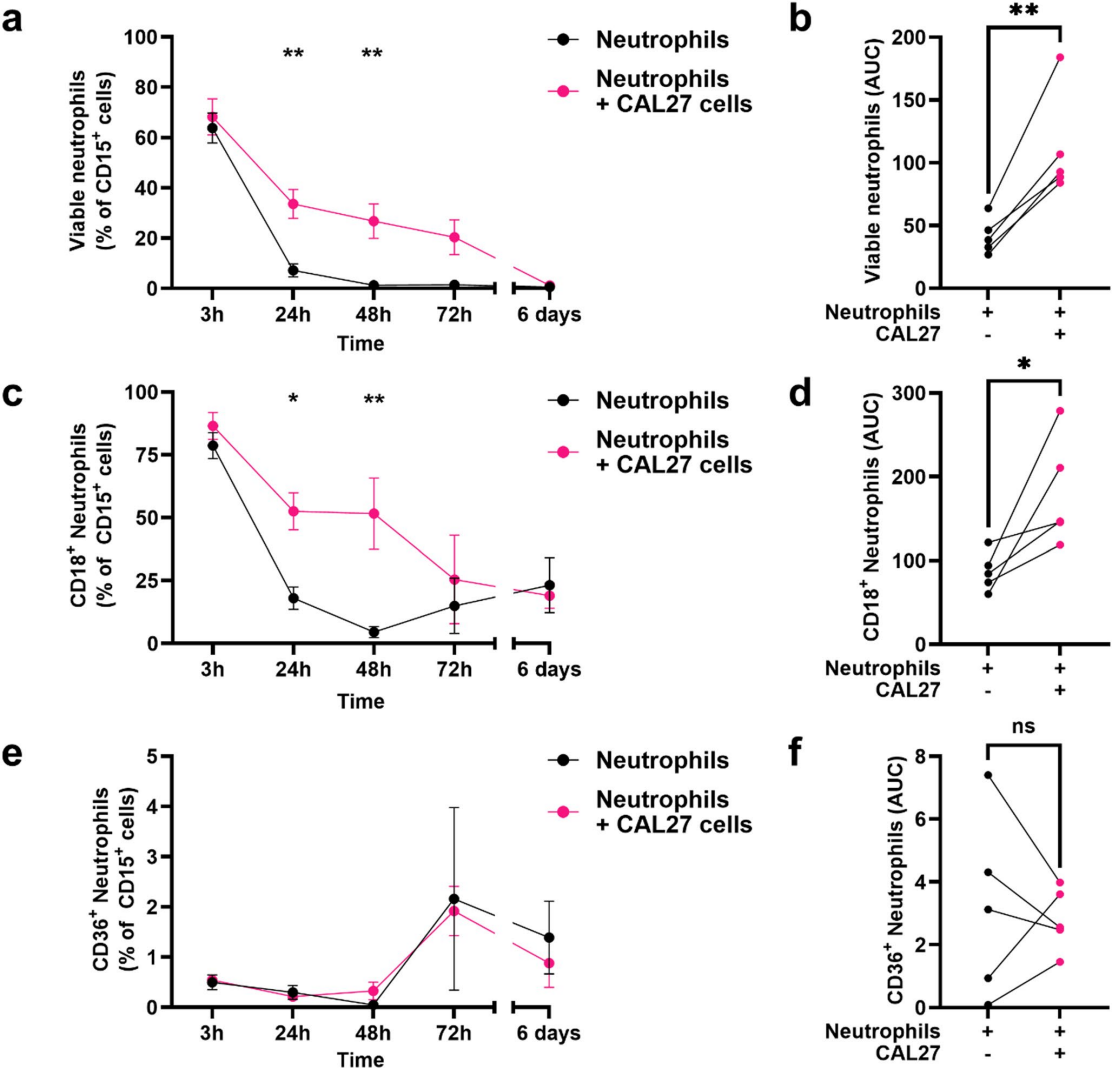
Temporal Analysis of Neutrophil Viability and CD18/CD36 Expression in Response to CAL27 Co-culture. Blood neutrophils from 5 healthy donors were cultured in vitro alone or together with CAL27 cells for 3, 24, 48, and 72 h (h), as well as 6 days. (**a**, **b**, **d**, **f**) Comparison of the percentage of (**a**) viable neutrophils, (**c**) CD18 + neutrophils, and (**e**) CD36 + neutrophils between those cultured alone and those co-cultured with CAL27 cells at each time point by two-way ANOVA followed by Bonferroni’s multiple comparisons test. (**b**, **d**, **f**) Comparison of the area under the curve (AUC) of the percentage of **(b**) viable neutrophils, (**d**) CD18 + neutrophils, and **(f**) CD36 + neutrophils between those cultured alone and those co-cultured with CAL27 cells across time by paired Student’s t-test. Data across all panels are presented as mean ± SEM, with statistical significance indicated by *p < 0.05, **p < 0.01

Neutrophils isolated from healthy donor blood were cultured with the oral cancer cell line CAL27 in vitro to investigate its impact on neutrophil lifespan and expression of CD18 and CD36. It became apparent that survival of neutrophils was markedly enhanced when co-cultured with CAL27 cancer cells, as evidenced by the existence of an enduring FSC-A^hi^SSC-A^hi^ population even after 6 days of culture (Fig. 2). Moreover, the percentage of viable neutrophils (PI^-^Annexin V^-^) was significantly higher at the 24 h (*p* = 0.0012) and 48 h (*p* = 0.0019) timepoints when co-cultured with CAL27 cells than when cultured alone (Fig. 3a). A similar pattern was observed at the 72 h timepoint though it failed to reach statistical significance. Finally, analysis of the cumulative survival of neutrophils across all timepoints revealed an overall higher viability in neutrophils co-cultured with CAL27 cells when compared to the control group (Fig. 3b).

Although the percentage of CD18^+^ viable neutrophils decreased during the duration of the experiment in both groups, co-culture with CAL27 cells resulted in a higher percentage of CD18^+^ viable neutrophils at the 24 h (*p* = 0.0142) timepoint compared to the control group (Fig. 3c). Moreover, area under the curve (AUC) analysis further confirmed an overall higher percentage of CD18^+^ viable neutrophils when co-cultured with CAL27 cells than when cultured alone during the study (Fig. 3d). In contrast, co-culture with CAL27 cells had no significant impact on the percentage of CD36^+^ viable neutrophils at any timepoint (Fig. 3e-f).

## Discussion

In the present study, we found that the surface levels of CD18 were higher in neutrophils from OSCC tumors and TDLNs than in those from hLNs, and that these elevated levels of CD18 were particularly evident in neutrophils from tumors of patients with metastatic OSCC. Notably, CD18 mediates adhesion to endothelial cells via ICAM-1, and thus plays a key role in neutrophil migration [15]. Nevertheless, ICAM-1 is also expressed by lymphocytes, and CD18-mediated contact has been implicated in the neutrophil-dependent suppression of T cell responses [11]. Moreover, expression of ICAM-1 has been reported in OSCC cells [16], and thus CD18 could also be essential for tumor-neutrophil crosstalk. In agreement with this, we found that co-culture of blood neutrophils from healthy donors with an oral cancer cell line resulted in prolonged neutrophil lifespan and sustained CD18 expression. Taken together, our results and those of others [17] suggest that the microenvironments of different tumors, such as breast cancer, and TDLNs drive an upregulation of CD18 in neutrophils, which might be essential to neutrophil-mediated suppression of anti-cancer immune responses.

In this context, the importance of TDLNs cannot be underestimated. TDLNs are critical sites for the initiation and modulation of tumor-specific immune responses, which can be impaired by metastasis in lymph nodes. The relationship between tumor size, lymph node metastasis, and neutrophil phenotypes is highlighted in previous work from this study, which mentions that both tumor size and lymph node metastasis directly impact the number and phenotype of circulating neutrophils. This, in turn, influences survival in patients with HNSCC [14, 18, 19]. Furthermore, research into neutrophil markers like CD18 indicates that these markers are upregulated in neutrophils from OSCC tumors, particularly in metastatic cases, providing further evidence of their relevance to tumor progression [20]. For these reasons, our findings on CD18 expression and association with cancer metastasis motivate further research into the potential role of CD18 in neutrophil immunomodulatory functions.

Although the present study demonstrates an upregulation of CD18 and CD36 expression in neutrophils from OSCC tumors and TDLNs, the underlying mechanisms by which these markers contribute to immune evasion are not fully elucidated. CD18, as an integrin β2 subunit, is know to facilitate neutrophil adhesion to endothelial cells via ICAM-1 [21], thereby promoting their migration into tumor sites and potentially enhancing their immunosuppressive activity [18]. This process has been implicated in immune evasion in cancers like lung squamous cell carcinoma [10]. Additionally, CD36, a class B scavenger receptor, has been linked to lipid metabolism and inflammatory signaling in immune cells. Increased CD36 expression in neutrophils within the tumor microenvironment may reflect adaptive mechanisms related to lipid-driven immune suppression, as observed in other cancers [12].These findings suggest that both CD18 and CD36 may contribute to neutrophil-mediated immune suppression, although further studies are needed to confirm these mechanisms in OSCC.

We chose CD18 and CD36 due to their established roles in neutrophil biology and tumor immunity. CD18 is a β2 integrin subunit essential for neutrophil adhesion and migration, particularly in the context of cancer progression, where neutrophils play a pivotal role in immune modulation and metastasis. CD36, a lipid transporter, has also been implicated in regulating the metabolic reprogramming of neutrophils within the tumor microenvironment, where it may facilitate immune suppression [22]. Although CD18 functions as part of the heterodimeric β2 integrins with various α subunits (αL, αM, αX, αD), our study focused specifically on CD18 because it is a common β subunit across these integrins and can serve as a representative for β2 integrin expression [15]. The decision was also influenced by the availability of antibodies targeting CD18. While further studies examining specific α subunits could provide more nuanced insights into integrin functions, CD18 remains a key marker for evaluating neutrophil trafficking and its involvement in tumor immunity. Unlike CD18, we found that the surface expression of the lipid transporter CD36 is only increased in neutrophils from OSCC tumors, but not in TDLNs. In addition, our in vitro experiments indicate that co-culture with an oral cancer cell line is not enough to induce the expression of CD36 in blood neutrophils from healthy donors. Taken together, these findings suggest that CD36 expression in neutrophils might require cellular interactions and signals that are unique to the in vivo tumor microenvironment. Importantly, we observed a particularly high expression of CD36 in neutrophils from tumors of patients with metastatic OSCC. In agreement with this, previous studies indicate that immunosuppressive neutrophils (i.e., PMN-MDSCs) favor lipid metabolism over glycolysis, and thus a number of lipid transporters—including CD36—have been forwarded as potential markers of PMN-MDSCs [12, 13]. Therefore, our results implicate CD36 in neutrophil-mediated immunosuppression and support the potential use of CD36 as a marker of PMN-MDSCs.

In summary, we have characterized the differential expression of CD18 and CD36 in neutrophils from two relevant anatomical compartments: the tumor microenvironment and TDLNs, and we have identified an association between the expression of these markers on neutrophils and the presence of metastasis in OSCC patients. Moreover, our results also demonstrate a direct effect of cancer cells on neutrophil survival and marker expression. Taken together, our findings could open new avenues for improving cancer immunotherapy. Specifically, targeting the pathways involved in the upregulation of CD18 and CD36 might inhibit the pro-tumorigenic functions of neutrophils, thereby limiting tumor progression and metastasis. Furthermore, the observed modulation of CD18 and CD36 also shows biomarker potential that could be used to determine prognosis and select therapeutic interventions aimed at curbing metastasis in OSCC. This connection between immune cell dynamics and cancer biology also offers a promising avenue for advancing personalized medicine in oncological treatments. Ultimately, this study motivates further research into the roles of specific neutrophil markers in cancer progression.

## Electronic supplementary material

Below is the link to the electronic supplementary material.

Supplementary Material 1

## Data Availability

No datasets were generated or analysed during the current study.

## Abbreviations

HNSCC: Head and neck SCC
hLN: Healthy lymph nodes
OSCC: Oral squamous cell carcinoma
PMN-MDSC: Polymorphonuclear myeloid-derived suppressor cells
ROS: Reactive oxygen species
SOC: Standard of care
TDLN: Tumor-draining lymph node
