# Supplementary material for: CD18 and CD36 expression in neutrophils from tumors and tumor-draining lymph nodes: implications for metastasis in oral squamous cell carcinoma
Source: Clin Exp Metastasis. 2025 Jun 23;42(4):37. doi: 10.1007/s10585-025-10356-z (PMC12185613; doi:10.1007/s10585-025-10356-z)
Supplement: Supplementary file 1 — Supplementary Material 1 [file 10585_2025_10356_MOESM1_ESM.docx]

| ANTIGEN | SUPPLIER/REFERENCE | RRID | CLONE | FLUOROCHROME |
| --- | --- | --- | --- | --- |
| CD15 | BD/563142 | AB_2738026 | W6D3 | BV711 |
| CD16 | BD/560474 | AB_1645561 | 3G8 | V450 |
| CD36 | BD/550956 | AB_398480 | CB38 | APC |
| CD45 | BD/566041 | AB_2744399 | HI30 | APC-R700 |
| CD18 | BD/749439 | AB_2873807 | L130 | BUV805 |

Supplementary Table 1. List of antibodies used in FACS analysis.


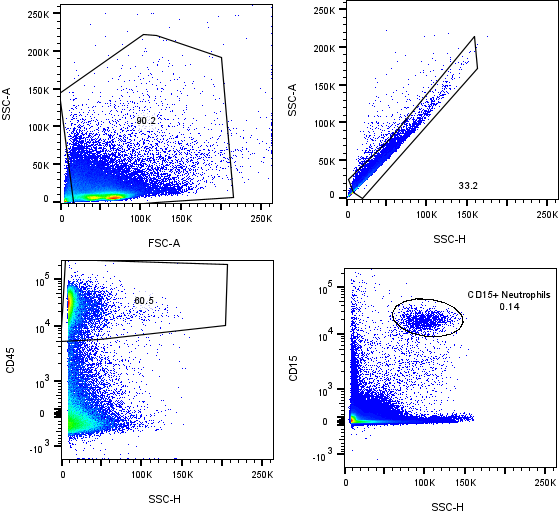


Supplementary Figure 1. The gating strategy for neutrophil identification in tumor and TDLN.

Supplementary Figure 2. The gating strategy for neutrophil identification in supernatant after co-culture with CAL 27 cells.
